# Development and evaluation of an innovative grain cart with a pneumatic conveyor

**DOI:** 10.1016/j.heliyon.2021.e08461

**Published:** 2021-11-24

**Authors:** Mohamed M. El-Kholy, Reham M. Kamel

**Affiliations:** Agricultural Engineering Research Institute, Agricultural Research Center, Giza 12611, Egypt

**Keywords:** Grain cart, Transportation cost, Performance test, Manufacturing, Innovation design

## Abstract

In line with the requirements of the Egyptian government to find a solution for wheat transportation during the peak harvesting season, an innovative design for a grain cart with a capacity of 8 tons supplemented with a grain hopper, a lifting double-action pneumatic conveyor, and a built-in digital scale was tested and evaluated to facilitate the transport of wheat crops from farmers' fields to storage sites. The cart was manufactured in the workshop of a local industrial company. It was tested under varying operational conditions in different wheat production areas in terms of working performance, efficiency of the grain loading and unloading mechanism, precision of the grain weighing mechanism, and cost/ton. The cart will enable wheat farmers and traders to transport and deliver their crops easily to storage sites with minimal losses and maximum working efficiency. It will also increase farmers’ profit because transportation cost by using this grain cart is less than that of the current conventional method. Moreover, the developed cart can secure wheat supply in government storage sites without any interruption during the peak wheat harvesting season.

## 1 Introduction

Wheat (*Triticum aestivum* L) is the most commonly cultivated cereal crop in the world. In Egypt, wheat is considered a strategic commodity and a key component of the people's diet. Egypt also has one of the highest levels of per capita wheat intake in the world (Curtis and Halford, 2014). Egypt ranks among the top five wheat importers worldwide, in accordance with the Food and Agriculture Organization. This enormous import amount places a considerable burden on the government's budget. Thus, alternatives should be considered to reduce the food gap in wheat.

Wheat is traditionally transported in private rented trucks (capacity: 6 tons) or pickup trucks (capacity: 2.5–3 tons) with a cost of approximately 60 LE (US$ 3.84) per ton to the Egyptian government's storage sites as the largest purchaser of domestic wheat crop. The government of Egypt has been trying to encourage wheat cultivation by increasing the procurement price to approximately 4833 LE (US$ 307.8) per ton. In most cases, private trucks take a long time to deliver wheat due to overcrowding at storage sites and may have to wait for more than 1 day, duplicating transportation costs. In addition, the manual packing/unpacking of wheat grains in temporary bags for transportation and the manual loading/unloading of grains in case of bulk transportation impose additional time and money losses that decrease the profit of farmers and storage sites. Consequently, many farmers agree to sell their crops on the field to traders with a 10% price decrease to avoid the inconvenience. All the aforementioned factors result in reduced amounts of domestic crops delivered to the government, which, in turn, forces the government to import wheat to satisfy the national demand for subsidized bread.

Several methods are used to transport and convey crops from fields to storage sites. The selection of the transporting method depends on the nature of application and the type of material being transported. Agricultural materials may be granular, powder, fibrous, or any combination of these materials (Barbosa-Cánovas et al., 2005). In general, conveying is accomplished via a combination of mechanical, inertial, pneumatic, and gravity forces (Duroudier, 2016; Hilton and Cleary, 2011; Owen and Cleary, 2010; Paliwal et al., 1999; Patel et al., 2013a, 2013b).

One problem with grain carts is loading grains into the cart. Heavy bulk bags, for example, must be mechanically loaded with a forklift, crane, or a similar device. Such process may be impractical in a field, and at best, inconvenient for many other procedures. In case of more traditional carts, loose seeds typically have to be filled from above the cart, or if from ground level through a separate belt or auger-type conveyor attached to seed sources, such as storage bins and trucks. However, these conveyors are not always easily available, especially for field operations. Moreover, an improved cart with a conveyor that can be used to load and unload seeds is evidently required (Chiu et al., 2019; Hafez et al., 2021). The current study aims to improve wheat transportation through the design, fabrication, and testing of an innovative cart for transporting wheat from fields to storage sites. The innovative design may differ from most available imported designs in terms of its pneumatic grain lifting and discharging mechanism, availability of a load cell for in-field grain weighting, and availability of a grain purity apparatus for assessing grain grades inside storage sites.

## 2 Materials and methods

### 2.1 Preparation of grain samples

The wheat variety Gemmiza-9 was selected for the experimental work on the basic of recent coverage area and the expected future expansion of planting this variety. Samples of the selected cultivar were collected at a moisture content of 12% w.b. and used for the experimental work during the 2019–2020 wheat harvesting season. The physical, mechanical, and aerodynamic properties of the wheat cultivar (cv. Gemmiza-9) are provided in Table 1.

**Table 1:** Physical, mechanical, and aerodynamic properties of wheat (Gemmiza_9).

Properties of wheat		Mean	Maximum	Minimum
Grain dimensions	Length, mm	7.32	7.39	7.29
	Width, mm	3.66	3.68	3.5
	Thickness, mm	2.90	2.92	2.89
1000-grain weight, g		47.54	49.83	45.70
Shape index, k		2.25	2.26	2.22
Coefficient of contact surface (C.C), %		60.39	60.43	60.29
Projection area, mm^2^		21.68	22.03	21.06
Bulk density, kg/m^3^	Loosely fill	815.51	830.22	808.59
	Vibrated fill	875.55	886.09	861.62
Porosity, %	Loosely fill	42.96	43.28	41.22
	Vibrated fill	40.14	41.12	41.00
Shear force in longitudinal direction, N		50.49	52.89	47.02
Shear force in lateral direction, N		45.09	48.97	42.69
Hardness, N		50.44	54.33	48.16
Friction coefficient	Galvanized sheet	0.33	0.34	0.32
	Iron sheet	0.40	0.44	0.38
	Painted iron	0.38	0.41	0.37
	Perforated iron	0.50	0.51	0..49
	Rubber sheet	0.47	0.48	0.44
	Stainless steel	0.25	0.26	0.22
	Wire net sheet	0.45	0.49	0.41

### 2.2 Description of the grain cart

**Figure 1:**
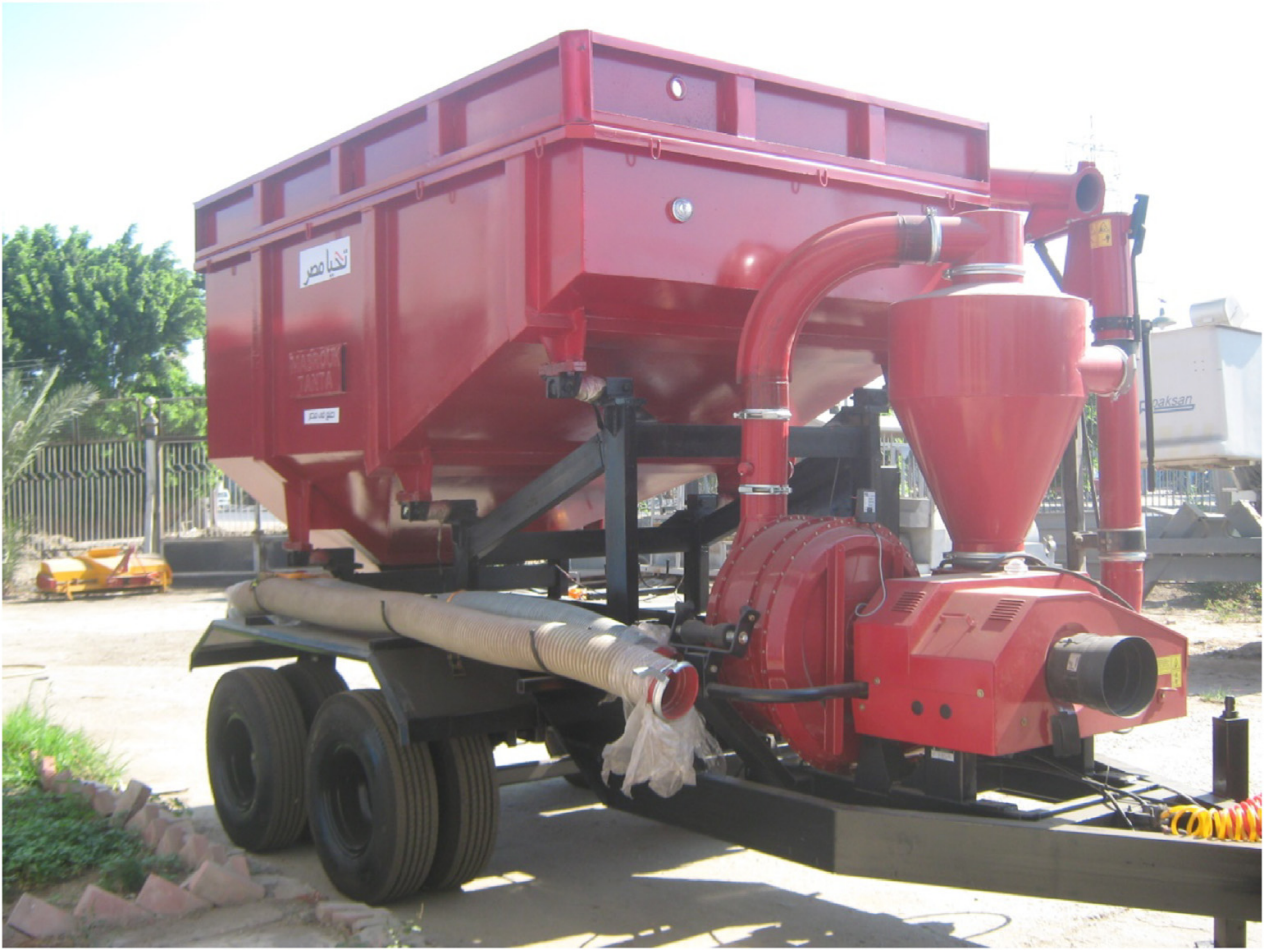
Photo of the developed grain cart for wheat transportation.

A grain cart with a pneumatic conveyor and an in-field weighting system was designed, fabricated, and tested for transporting wheat grains from field locations to storage sites as shown in Figure 1. The specifications and materials used in the cart components are provided in Table 2. Meanwhile, the mechanical and physical properties of the individual component materials are presented in Table 3 (Shao et al., 1998; Dawson, 1999). The major components of the grain cart included the following: 1) chassis and axle system; 2) grain hopper, 3) pneumatic conveyor, 4) weighting and recording system, and 5) grain moisture and purity assessment system.

**Table 2:** Components and materials used in the grain cart structure (Clause 5.1).

SI No. (1)	Components (2)	Materials (3)	Refer to IS no. (4)	Grade (5)
i.	Chassis	Mild steel	IS 2062	Fe 410-S
ii.	Drawbar	Mild steel	IS 2062	Fe 410-S
iii.	Two eyes or two jaws	Carbon steel	IS 1875 or IS 1030	20 C Grades 23 to 45
iv.	Platform	Mild steel	IS 2062	Fe 410-S
v.	Sideboards	Mild steel Timber	IS 2062 IS 2179	Fe 410-S
vi.	Leaf spring	Spring steel	IS 3431	***ـــــــــــ***
vii.	Axle	Structural steel	IS 2062	Fe 410-S
viii.	Hub	Cast iron	IS 210	FG 200
ix.	Brake drum	Cast iron	IS 210	FG 200
x.	Cap	Cast iron Mild steel	IS 210 IS 1079	FG 200 ***ـــــــــــــ***
xi.	Washer	Mild steel	IS 2062	***ـــــــــــــ***

∗(Indian standard, 2000).

**Table 3:** Mechanical and physical properties of the individual components of the grain cart.

Materials	Mild steel	Mild steel	Carbon steel	Cast iron
Refer to IS no.	IS 2062	IS 1079	IS 1030	IS 210
Physical properties				
Mass density, g/cm^3^	7.73	7.90	7.85	7.15
Melting point, °C	1480–1530	***ــــــ***	1510	1127–1204
Modulus of elasticity, GPa	200	265	210–190	92.4
Specific heat capacity, kJ/kg. °K	0.46	0.32	0.44	0.46
Thermal expansion coefficient (15–75°C), μm/m °C	11	***ـــــــ***	11.7	10.5
Thermal conductivity, W/m °K	26.9	31.3	51.9	20–80
Mechanical properties				
Yield strength, MPa	290	326	440	***ـــــــ***
Tensile strength, MPa	444	468	525	207
Elongation, %	33	41	32	13

∗(Shao et al., 1998; Dawson, 1999).

#### 2.2.1 Chassis and movement system

**Figure 2:**
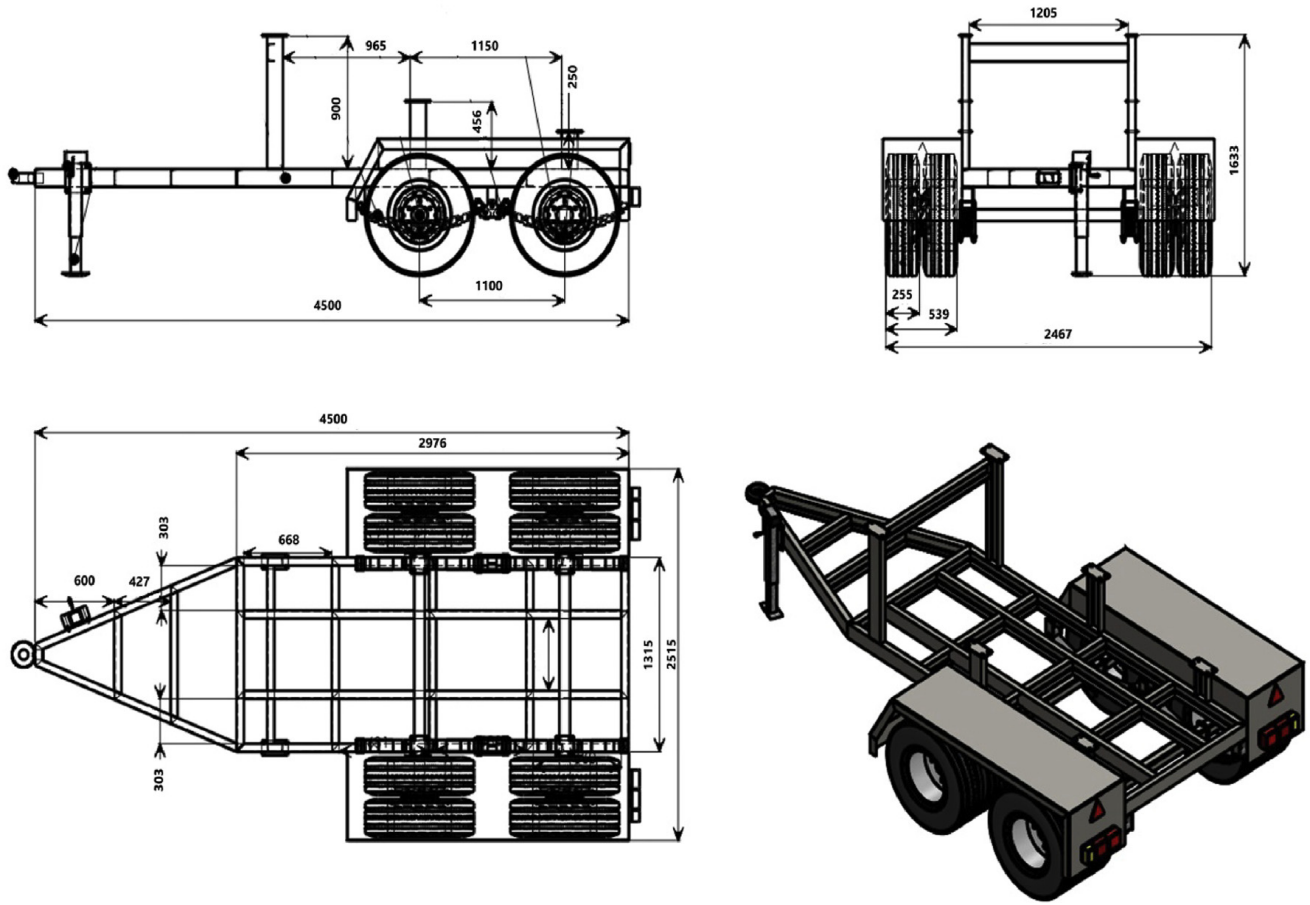
Perspective view of the grain cart showing the internal structure of the frame and the attachment method to the tractor.

**Figure 3:**
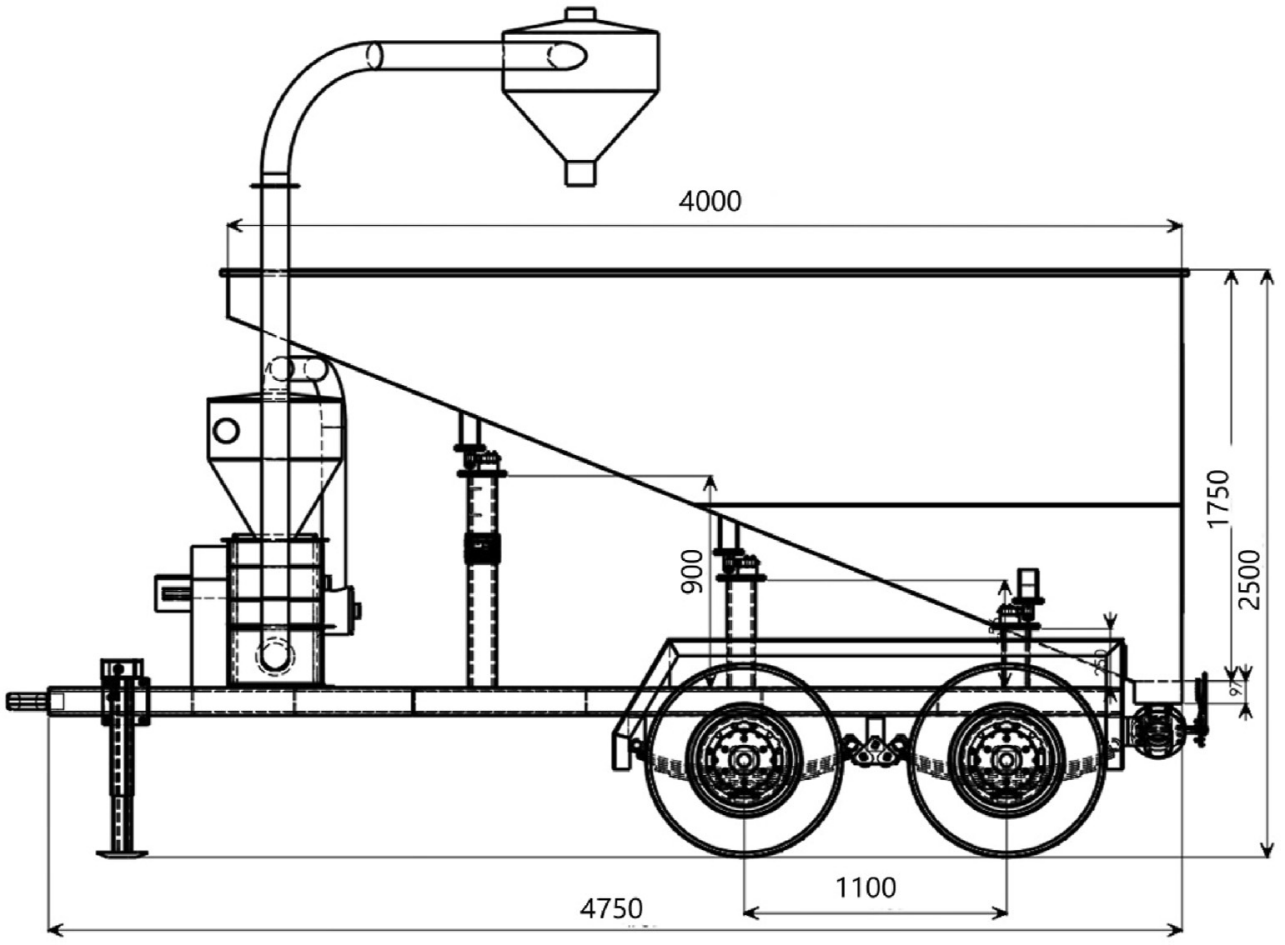
Side view of the grain cart illustrating the dimensions of the grain hopper, and chassis and the distances between the wheels.

The cart chassis was designed to work in non-paved areas in villages (Figures 2 and 3). The chassis dimensions were 4.5 m (length) and 2.5 m (width). The chassis was manufactured from high-quality steel sectors (UPN) and could bear up to 8 tons grain capacity added to the wagon weight. Four spring iron sliders (1.0 m long/spring) were used for suspending the chassis over the cart wheels. Each slider consisted of 12 iron chips with a width of 120 mm and a thickness of 10 mm (Tundam system) suitable for incident loads while keeping the lower level of the cart over the ground for convenient movement and stability. Eight rubber wheels' with a size of 15/12.5 (or the equivalent) were used. The inner distance between a wheel's axis was 1.10 m, while the outer distance was 2.46 m. The cart was equipped with a hydraulic brake system.

#### 2.2.2 Grain hopper

**Figure 4:**
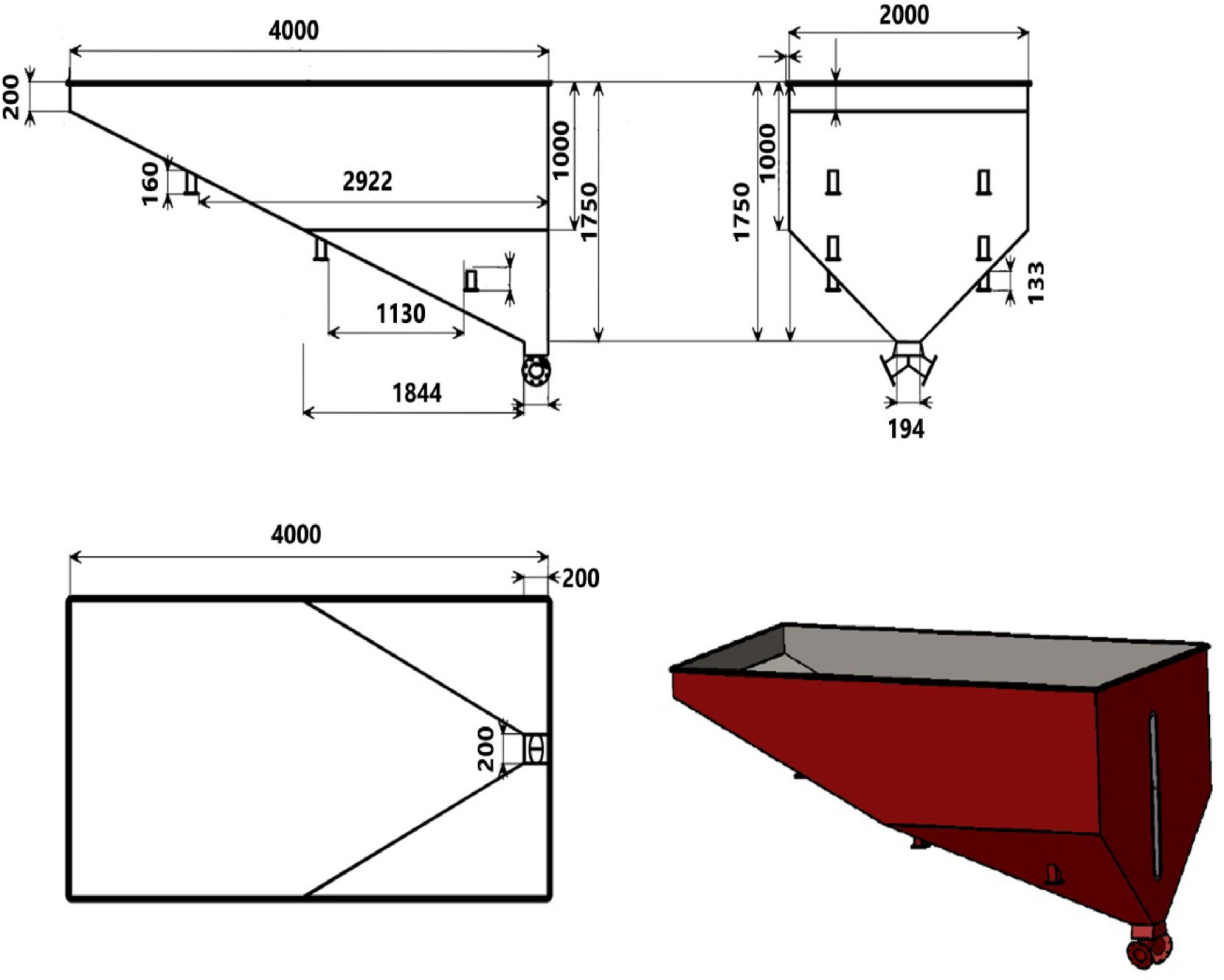
Fragmented schematic details of the grain hopper with the dimensions of different parts and the grain discharge gates at the rear of the hopper.

A grain hopper generally consists of an upper part with structurally substantial vertical sidewalls (Figure 4). In the developed cart, the grain hopper was supported by a pair of rectangular frames. The hopper had a lower part with three triangular wall extensions. Each extension tapered inward and downward to converge at an outlet that opened into a rectangular opening discharge with a hinged spring gate at the center and two circular openings at the sides. Conventionally, this structure directs all seeds loaded into the hopper toward the discharge points.

#### 2.2.3 Pneumatic conveyor

**Figure 5:**
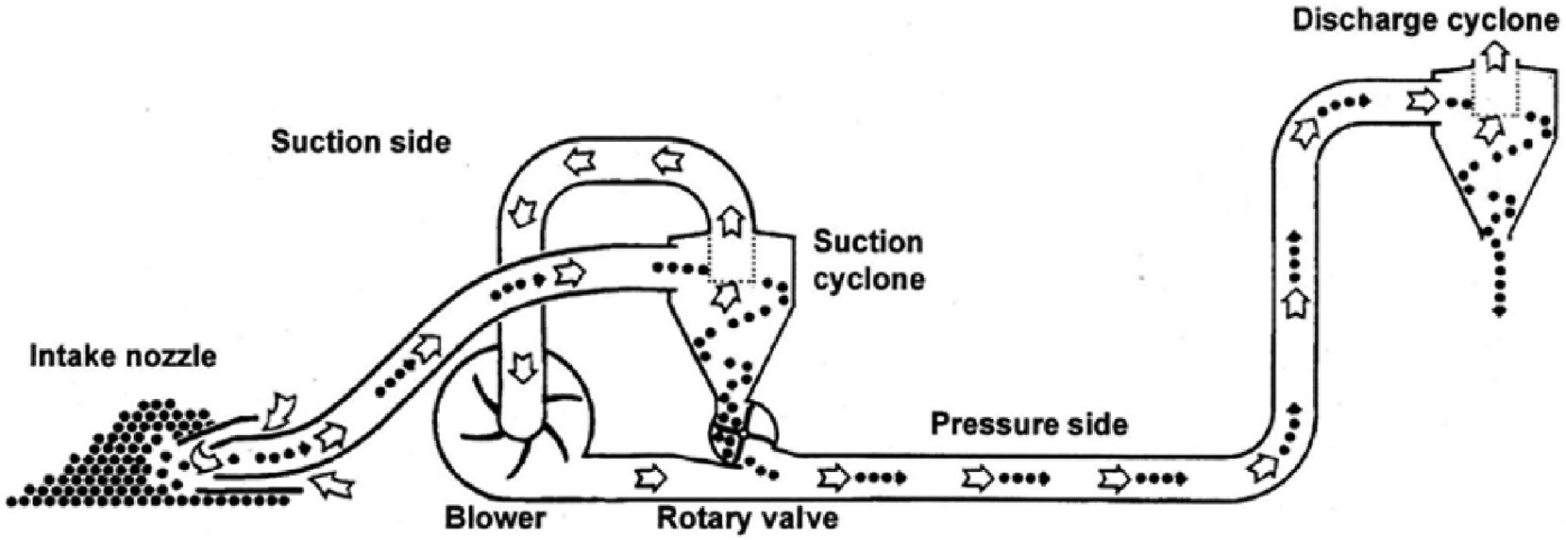
Illustration of the pneumatic conveyor used for loading and unloading grains.

The suction blower of the pneumatic conveyor consisted of a powerful blower and a rotary valve, as shown in Figure 5. Conveying was initiated using the suction air of the blower to lift and accelerate grains toward the blower. Before the grains reached the blower housing, they were separated from the air stream in a cyclone and dropped into the rotary valve. Meanwhile, the air continued to move through the blower. The rotary valve created an airlock that conveyed grains from the suction side in the cyclone to the pressure side in the transport pipe. The grains were then transported to the outlet cyclone in the air stream. To achieve a balance between the air and the material, the suction blower was equipped with an adjustable intake nozzle. The blower was provided with an automatic air regulator positioned in the pipe between the cyclone and the blower intake to limit the maximum air speed to approximately 25 m s^−1^. Thus, grain damage owing to the excessive speed and overloading of the blower was avoided.

The air regulator consisted of a spring-loaded butterfly valve that remained open when the blower was stopped. When blower operation started, the airflow closed the regulator, such that airspeed would not exceed approximately 25 m s^−1^. When back pressure dropped, the air regulator would close sufficiently to prevent airspeed from exceeding approximately 25 m s^−1^. The air regulator will only be effective if the spring of the regulator is properly adjusted. Therefore, the spring should not be adjusted unless proper equipment is used to check that the ideal conveying speed is maintained. If the spring of the air regulator is too slack, then the maximum airspeed is reduced, and thus conveying capacity is diminished. Simultaneously, risk of material settling in the pipes and plugging can occur.

If the spring is over-tightened, then the maximum airspeed is increased, intensifying the risk of damage to the conveyed products. Capacity is not increased but the blower and the tractor will be under more loads. Hence, the belts and bearings of the blower will be loaded over their designed limits, considerably shortening their life span.

#### 2.2.4 Grain weight and recording system

**Figure 6:**
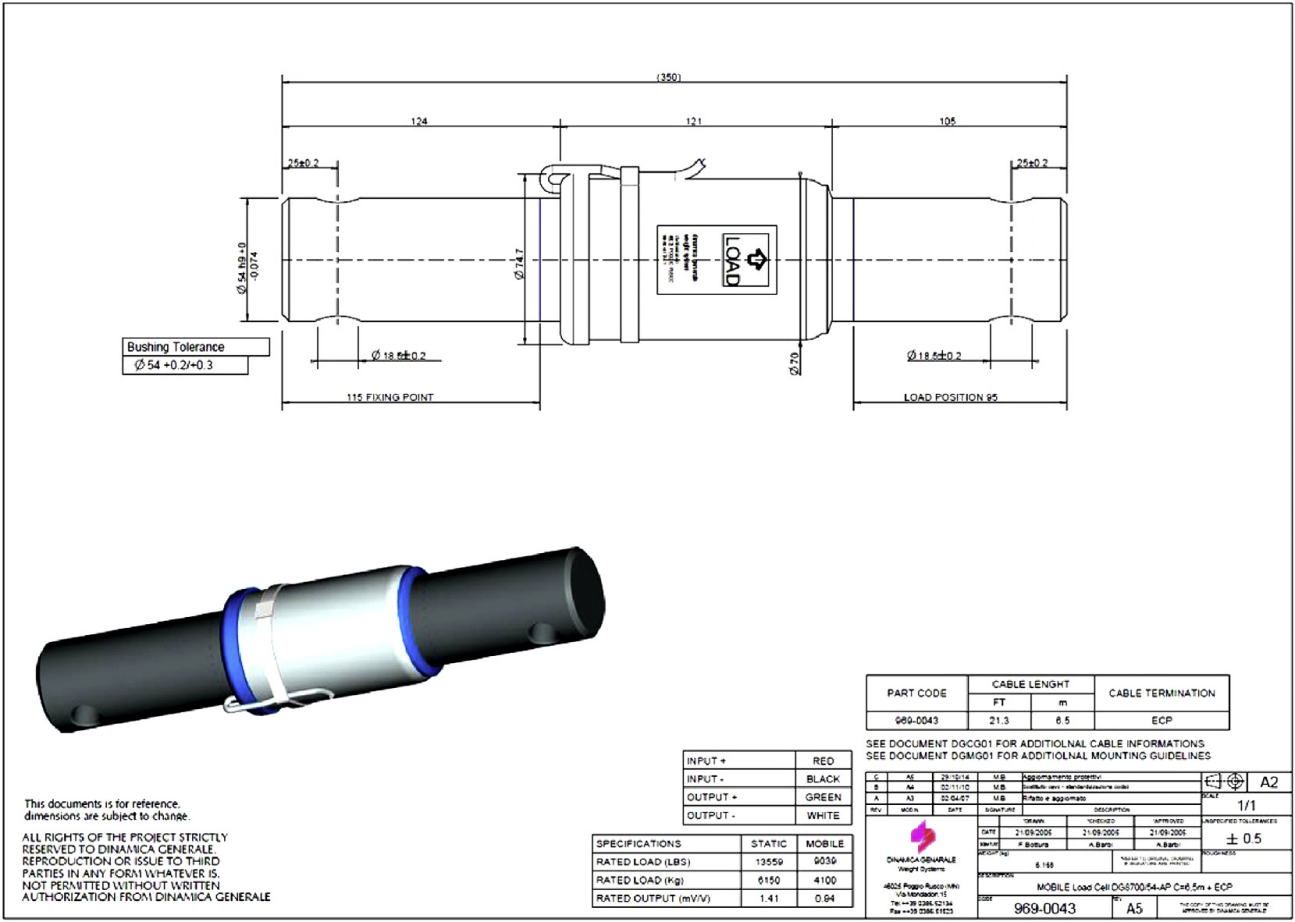
Feature of load cells used for the weighting system.

A precise load cell weighing system (Figure 6) with a recording unit was used to weigh grains directly in the field (accuracy: ± 1 kg). Four load cells were fixed over the frame of the grain cart and connected to a high-tech indicator. The position of the load cells over the chassis frame of the grain cart was determined based on laboratory experiments to obtain the highest weight accuracy of the grain.

#### 2.2.5 Grain moisture content and grade assessment devices

A precise grain moisture meter and a grain impurity separation unit were included in the grain cart to determine grain moisture content and grain purity in the storage sites, respectively. The two indicators were used for assessing grain grade and price.

### 2.3 Testing and performance evaluation of the grain cart

The pneumatic conveyor of the grain cart was designed and manufactured in cooperation with POM Augustow Company in Poland. Preliminary tests for the working performance of the conveyor were conducted in POM Augustow Company to optimize the operational condition of the unit. The working efficiency of the conveyor material with different lengths of the suction section is presented in Table 4.

**Table 4:** Testing conditions of the pneumatic conveyor.

Test number	Work specification
1	Total suction section length 10 m polyvinyl chloride (PVC) hose 10 m
2	Total suction section length 3.3 m PVC hose 3.3 m
3	Total suction section length 6 m (2 × bend 900 + 0.3 m metal pipe) polyurethane (PUR) hose 3.3 m
4	Total suction section length 6 m (2 × bend 900 + 0.3 m metal pipe) PVC hose 3.3 m
5	Total suction section length 11 m (2 × bend 900 + 0.3 m + 2 × 2 m + 1 × 1 m metal pipes)
6	Total suction section length 10 m (2 × bend 900 + 0.3 m + 2 × 2 m metal pipes + bend 300) PVC hose 3.3 m

### 2.4 Field performance of the grain cart

The performance of the grain cart, including that of the pneumatic conveyor, was evaluated under Egyptian conditions. The experimental work was conducted during the wheat harvesting season (2019–2020) in Kafr El-Sheikh Governorate, Egypt. The testing procedure included the following steps.

#### 2.4.1 Performance of the pneumatic conveyor

The pneumatic conveyor was tested for grain loading from the field and grain unloading from the cart into storage sites (silo reception pits or permanent burlap bags) by using the grain discharging rear valve. The tractor was operated until the power take-off reached the appropriate velocity for the pneumatic conveyor as indicated through the velocity device attached to the pneumatic conveyor. Once achieved, the hose was connected to the suction pipe of the pneumatic conveyor, oriented towards the grain pile and the suction process continued until the cart was completely filled. A stopwatch was used to assess the charging time for each studied condition (silos/burlap bags). For the discharging process, the gate was adjusted and the time of emptying a cart was also determined. Each test was replicated three times, and the average of the readings was considered.

#### 2.4.2 Performance of the load cell weighing system

To assess the performance and accuracy of the load cell weighing system, three different amounts of grains (6500, 7000, and 7500 kg) were examined. The load cell system was compared with a standard digital balance (Model Scale-Tec POINT, USA) (accuracy: ± 0.1kg) and the Bascool balance (GROMY, Model no. GS-EA, China) (accuracy: ± 0.2 kg) as references.

#### 2.4.3 Grain cleaning during pneumatic dischargin***g***

The percentage of trash (g/hg of grains) was determined before and after the discharging process by using the pneumatic conveyor of the cart. Three grain samples were taken for laboratory analysis by using the trash separating unit (NORITAKE, model JP-11-R, Japan).

#### 2.4.4 Grain cart balance

Dynamical studies of grain cart stability were conducted during straight and turning movements (Abubakar et al., 2010; Ahmadi, 2013; Dechao et al., 1989). The stability of the developed grain cart was tested on different roads and turns during grain transportation from the collection site (Kafr El-Sheikh Farm) to a storage site in Qallin Village (25 km) and on unpaved roads of the Meet EL Dyba experimental farm when transporting grain from the combine harvester to the grain collection area. A four-wheel drive tractor (Belarus 160 HP) for moving the grain cart was used to evaluate cart balance.

**Figure 7:**
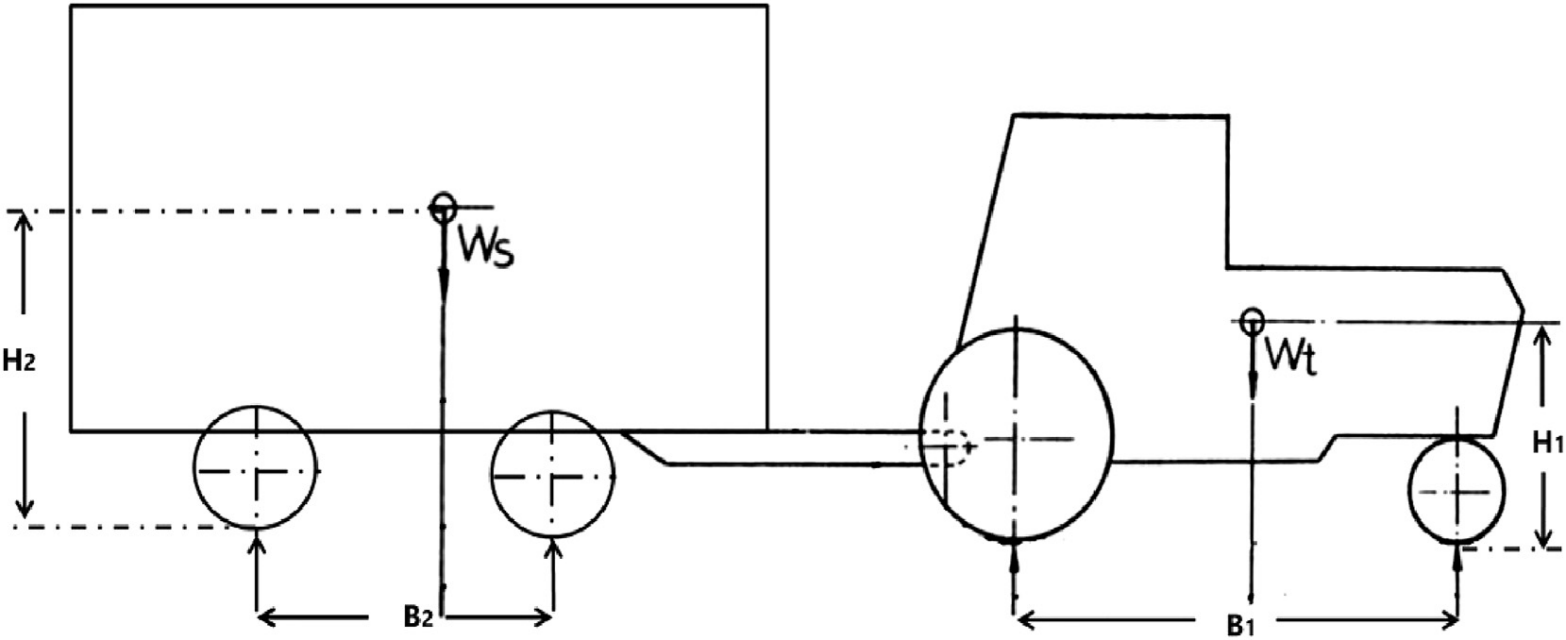
Center of gravity locations for the tractor grain cart.

The forces acting on the system included the weights of the tractor and trailer ($W_{t}$ and $\;W_{s}$, respectively) as shown in Figure 7. The horizontal wheel spaces of the tractor ($B_{1}$) and grain cart ($B_{2}$) were 2.546 m and 1.10 m, respectively. However, the variables of vertical distances from the surface of the terrain to the center of gravity of the tractor ($H_{1}$) and the grain cart ($H_{2}$) were 0.853 m and 1.670 m for the full loading case, respectively.

The overturned position of the combination was defined as follows: when the transverse rolling angle of either the tractor body ($\theta_{t}$) or the cart body ($\theta_{c}$) or both is greater than a threshold angle ($\theta_{v}$) (Dechao et al., 1989) as indicated in Table 5.where B is wheel spacing (m), and H is the center of gravity height (m) of the tractor or the grain cart.

**Table 5:** Conditions of stability for the tractor and the grain cart rolling angle.(1)$\theta_{v}=\pi \;arc\;\tan\;\left(\frac{2H}{B}\right),\;$

Position	$\theta_{t}$	$\theta_{c}$
Stable	0	0
Unstable	>0	$<\theta_{\nu }$
Overturned	$<\theta_{\nu }$	0
	$>\theta_{\nu }$	$<\theta_{\nu }$
	or $<\theta_{\nu }$	$>\theta_{\nu }$
	or $>\theta_{\nu }$	$>\theta_{\nu }$

### 2.5 Cost analysis of the grain cart

Fixed and operation costs of the developed grain cart and the tractor were estimated as reported by (Bochtis et al., 2019). Fixed costs included depreciation, interest, repairs taxes, and insurance. Depreciation is a cost that results from the wear, obsolescence, and age of a machine. The degree of mechanical wear may cause the value of a particular machine to be relatively above or below the average value for similar machines when it is traded or sold (Poozesh et al., 2012). Depreciation can be estimated using Eq. (2):

(2) $$Depreciation=\frac{PP-SV}{10},\;$$

where SV is the salvage value (10%) of the purchase price (PP). The *PPs* of the tractor and the grain cart were US$ 20700 and US$ 24840, respectively.

Interest cost is the reward paid by a borrower to a lender for the use of money for a certain period, as expressed in Eq. (3), and taxes and insurance costs are estimated to be equal to 10% of the purchase price (Robb et al., 1998).

(3) $$Interest=\frac{PP+SV}{2}\;\times \frac{i}{100},\;$$

where the average interest rate i is assumed to be 10%.

Operating costs included fuel, lubrication, labor, and maintenance. Fuel cost was estimated using the average fuel consumption for field operations as 10 L/h for 960 h, while the estimated lubrication costs averaged approximately 18% of fuel costs (Robb et al., 1998; Seyyedhasani, 2017). In addition, repair costs were estimated at 5%–8% of the purchase price as indicated by Bochtis et al. (2019). Thus, 6.5% of the total price was imposed as a maintenance cost for the current study.

## 3 Results and discussion

### 3.1 Performance of the pneumatic conveyor

As shown in Table 6, the wheat lodged inside the hose during grain conveying by the pneumatic conveyor with a connected 10 m−long PVC suction hose. Transport was possible, but efficiency was extremely low. Further tests were performed on different types of hose and other metal connections to determine the best operating conditions for hose structure, angle, and number of bends. A lower angle was desirable to prevent airway obstruction as indicated by Gao et al. (2020).

**Table 6:** Efficiency tests for the pneumatic conveyor T 449.

Test number	Weight	Time	Efficiency
	kg	s	t/h
1	--------	------	------
	Observation: Transport was possible, but wheat lodged in the hose. Hence, no measure of efficiency was determined		
2	1330	180	26.5
3	1480	270	19.7
4	1515	265	20.6
5	1480	275	19.3
6	1535	265	20.8

**Transported material:** wheat 700 kg m^−3^; moisture content: 12 % (w.b.).

A standard length of 3.3 m of the flexible suction hose confirmed high efficiency and a suction level of 26.5 ton h^−1^ (Test 2). Leaving the inlet slot with two bends and a section of straight metal pipe at a length of 300 mm (Tests 3 and 4) exhibited lower efficiency of the system (range: 19.5 ton h^−1^−20.8 ton h^−1^) and depended on using the appropriate type of hose (PVC or PUR). In the case wherein 5 m of the metal pipe was added (Tests 5 and 6), the efficiency of the system practically did not change and was on the level of 19.5 ton h^−1^ for the PUR hose and approximately 20.8 ton h^−1^ for the PVC hose. By comparison, between (Test 2) and (Tests 3–6), efficiency decreased due to the presence of bends. This result is similar to those of other studies (Das and Meloy, 2002; Klinzing et al., 1997), which indicated that bends in a pneumatic pipeline can be responsible for a considerable proportion of pressure drop and that the flow rate of grains becomes less as pressure drop increases. That is, the suction length of the 3.3 m PVC hose achieved the highest efficiency of 26.5 ton/h. However, the use of a 10 m−long flexible suction hose on the suction side is not recommended. To make the suction side longer, metal pipes are recommended because they will maintain the efficiency of grain transfer while avoiding pressure drop inside the tubes.

### 3.2 Grain loading and unloading capacity

As shown in Table 7, the cart's loading and unloading capacity approached average values of 25.87 ton h^−1^ and 26.38 ton h^−1^, respectively, when using the pneumatic conveyor system. However, the unloading capacity of the rear unloading valve was 27.81 ton h^−1^. That is, the cart with an 8 ton capacity can be loaded within 18.55 min and unloaded within 18.1 min by using the pneumatic conveyor. However, the cart was unloaded within 17.25 min when the rear valve was used for filling burlap bags. In general, airflow velocity does not exceed 3–15 m/s at the beginning of the conveying pipe and 25–50 m/s at the end of the path (Shishkin et al., 2020). This finding confirmed that grain loading capacity is always less than grain unloading capacity, which may be expected due to the effect of gravity.

**Table 7:** Performance tests of the grain cart under actual field conditions.

Test Item	Test 1	Test 2	Test 3	Average
**1-Pneumatic conveyor** Grain loading (ton h^−1^) Grain unloading (ton h^−1^)	25.79 26.35	25.95 26.38	25.89 26.42	25.87 26.38
**2- Rear valve discharge** (ton h^−1^)	27.35	28.12	27.98	27.81
**3- Grain weight** (kg) Standard digital balance Bascool balance Cart load cell weight system	6500 6436 6477	7000 6942 6978	7500 7451 7489	7000 6943 (−0.81%) 6981 (−0.27%)
**4- Cart balance**				
	${\text{θ}}_{\text{t}}=0.001$ ${\text{θ}}_{\text{c}}=0.003$	${\text{θ}}_{\text{t}}=0.000$ ${\text{θ}}_{\text{c}}=0.012$	${\text{θ}}_{\text{t}}=0.010$ ${\text{θ}}_{\text{c}}=0.002$	$\theta_{t}=0.003$ $\theta_{c}=0.006$
**5- Grain cleaning during discharge process (pneumatic)** % of trash (g/hg of grains) Before discharge After discharge Percentage of cleaning	0.9% 0.2% 78%	1.8% 0.4% 77%	2.3% 0.9% 60%	1.6% 0.5% 69%

### 3.3 Grain weight

The precision of determining grain weight by using the load cell system was evaluated and compared with using a standard digital balance and the Bascool balance. As indicated in Table 7, the grain weight of the cart load cells was less than the standard weight balance by approximately 0.27%, while the Bascool balance presented a lower weight by 0.81%. Thus, the load cell weighing system of the cart exhibited more precision than the Bascool balance.

### 3.4 Grain cleaning during the charging process

As indicated in Table 7, the average percentage of trash in the wheat samples decreased from 1.6% to 0.5% after passing through the pneumatic elevator during discharging. Thus, the pneumatic discharging mechanism can decrease the percentage of trash in wheat by approximately 69%. This phenomenon might have occurred on the side of the cyclone separator due to the effect of air passing through the grain during the unloading process, removing the trash of the lower terminal velocity from the unloading grain (Güner, 2007). The results are in agreement with those of Baker et al. (1986), who evaluated corn grain damage and dust production and reported that fines produced varied from 0.04% to 1.43% of the weight of the conveyed grains.

### 3.5 Grain cart balance

As indicated in Table 7, the average values of the rolling angle of the tractor ($\theta_{t}$) and the cart ($\theta_{c}$) were nearly zero. That is, the tractor and cart were in stable condition with good balance during the transportation of wheat from the farm area to the storage site (25 km distance). These results are in agreement with those of Dechao et al. (1989).

### 3.6 Cost analysis of grain cart

Various items for the cost analysis of the tractor and the grain cart were estimated and compared with the traditional method (pickup truck) as presented in Table 8. Farmers use pickup trucks to transport their wheat crops from fields to storage sites. The transportation cost per ton of wheat by using temporary bags with 50 kg capacity (20 bags/ton) with the price of US$0.096/bag was US$1.91/ton. In addition, labors’ wages for loading the bags in the field location and unloading them in the storage site were US$2.55/ton and truck rental cost was approximate US$3.84/ton. Thus, the total fixed and operation costs were estimated as US$10.38/ton after adding 25% profit.

**Table 8:** Cost analysis and profitability of the developed grain cart.

Cost item	Pickup truck	Tractor	Developed grain cart
Fixed cost			
Depreciation	∗Included in the rental price	US$1863.07	US$2235.68
Interest		US$1252.56	US$1502.88
Taxes and insurance		US$207.00	US$248.40
Total fixed cost		US$3322.63	US$3986.96
Total fixed cost/ton	**US$3.84/ton**	**US$0.65/ton**	**US$0.78/ton**
Operating cost			
Fuel	US$0	US$3312.12	US$0
Lubrication	US$0	US$611.47	US$0
Labor	Labors for charge and discharge (US$2.55/ton)	US$764.34	US$573.25
Repair and Maintenance		US$186.31	US$223.57
Others	Burlap bags (US$1.91/ton)		
Total operation cost		US$4874.23	US$796.82
Total operation cost/ton	**US$4.46/ton**	**US$0.96/ton**	**US$0.16/ton**
Total fixed and operation cost/ton		**US$1.61/ton**	**US$0.94/ton**
	**US$8.3/ton**	1.61 + 0.94 = **US$2.55/ton**	
To achieve a profit of 25% for the trader, total cost became	**US$10.38/ton**	**US$3.19/ton**	

∗ Assumption: $5.3\frac{ton}{h}\times \frac{16h}{day}\times \frac{60day}{season}=5088\;ton/season$

When the purchase price of the tractor was US$20700 and the cost of manufacturing the cart was US$24840, the total fixed costs for both were estimated as US$0.65/ton and US$0.78/ton, respectively. Meanwhile, the operating cost for the process of transferring wheat included labor wages, which were estimated for 60 days (the duration of the wheat harvest season), fuel, lubrication, and repairs for the tractor and the grain cart, which was estimated as US$1.12/ton. Thus, the total cost (US$3.19/ton) was less than 1/3 of the cost of the traditional method (US$10.38 $/ton).

## 4 Conclusion

The developed grain cart demonstrated good working performance and more advantages over the current commercial wheat transportation methods. The innovative pneumatic conveyor system can load and unload the developed cart without the need for additional machines or labor. Moreover, the weight load cells can accurately weigh grains in the field, allowing for simultaneous wheat collection from different farms. The pneumatic system used for loading and unloading the cart can also separate dust and trash from the grains, eliminating the necessity for this step in storage sites. In general, the application of the developed grain cart at a commercial scale will help farmers sell their wheat crops directly from the field with low transportation costs and precise grain weight. In addition, the cart will facilitate the delivery of a high-quality wheat supply to government storage sites and help accomplish the goal of providing food security in Egypt.

## Declarations

### Author contribution statement

Mohamed M. El-Kholy: Conceived and designed the experiments; Performed the experiments; Contributed reagents, materials, analysis tools or data; Wrote the paper.

Reham M. Kamel: Performed the experiments; Analyzed and interpreted the data; Contributed reagents, materials, analysis tools or data; Wrote the paper.

### Funding statement

This work was supported by the Academy of Scientific Research and Technology Fund, Egypt (Project ID: 10334).

### Data availability statement

Data will be made available on request.

### Declaration of interests statement

The authors declare no conflict of interest.

### Additional information

No additional information is available for this paper.
